# Genome conformation capture reveals that the *Escherichia coli* chromosome is organized by replication and transcription

**DOI:** 10.1093/nar/gkt325

**Published:** 2013-04-30

**Authors:** Cedric Cagliero, Ralph S. Grand, M. Beatrix Jones, Ding J. Jin, Justin M. O’Sullivan

**Affiliations:** ^1^Gene Regulation and Chromosome Biology Laboratory, Frederick National Laboratory for Cancer Research, National Cancer Institute, National Institutes of Health, Frederick, MD 21702, USA, ^2^Institute of Natural and Mathematical Sciences, Massey University, Auckland 0745, New Zealand and ^3^Liggins Institute, University of Auckland, Auckland 1023, New Zealand

## Abstract

To fit within the confines of the cell, bacterial chromosomes are highly condensed into a structure called the nucleoid. Despite the high degree of compaction in the nucleoid, the genome remains accessible to essential biological processes, such as replication and transcription. Here, we present the first high-resolution chromosome conformation capture-based molecular analysis of the spatial organization of the *Escherichia coli* nucleoid during rapid growth in rich medium and following an induced amino acid starvation that promotes the stringent response. Our analyses identify the presence of origin and terminus domains in exponentially growing cells. Moreover, we observe an increased number of interactions within the origin domain and significant clustering of SeqA-binding sequences, suggesting a role for SeqA in clustering of newly replicated chromosomes. By contrast, ‘histone-like’ protein (i.e. Fis, IHF and H-NS) -binding sites did not cluster, and their role in global nucleoid organization does not manifest through the mediation of chromosomal contacts. Finally, genes that were downregulated after induction of the stringent response were spatially clustered, indicating that transcription in *E. coli* occurs at transcription foci.

## INTRODUCTION

Our understanding of the spatial organization of bacterial genomes and its relationship to cellular function is limited [for reviews see (1–3)]. Yet it is clear that despite not being enclosed in a nuclear membrane, bacterial nucleoids are spatially organized within a defined sub-fraction of the cell volume (4–11). Various molecular [reviewed in (2)] and recombination-based methodologies have been used to identify the existence of micro- and macrodomains within the *Escherichia coli* nucleoid [e.g. (2,5,8,12,13)]. The four structured macrodomains (∼0.5–1 Mb) that have been identified exhibit preferential intra-domain recombination between λ att sites, whereas inter-domain recombination is reduced (5,7,8,12,13). By contrast, microdomains are much smaller (average ∼10 kb) and have been linked to the topological isolation of supercoils (2,10). Collectively, micro- and macrodomains are hypothesized to be critical for maintaining global organization while enabling the local levels of compaction required to fit a circular chromosome with an extended diameter of ∼490 nm within a cell with a length as small as 1000 nm (2).

Unlike eukaryote chromatin, the bacterial nucleoid does not contain histones. However, nucleoid-associated proteins (NAPs), particularly histone-like proteins, such as histone-like nucleoid structuring (H-NS) protein, heat unstable nucleoid protein (HU), factor for inversion stimulation (Fis) and integration host factor (IHF), are believed to act like histones and play a significant role in the organization of the nucleoid (14–17). These NAPs exhibit DNA bending, looping and bridging properties *in vitro.* However, studies also indicate that *in vivo*, the role of the NAPs could be more regulatory than architectural [e.g. (18,19)]. Non-classical NAPs (i.e. SeqA, SlmA and MatP) have been recently characterized as exhibiting macrodomain-specific DNA-binding properties [reviewed in (16)] and may represent alternative candidates for organizational roles within the nucleoid.

The structure of the bacterial nucleoid is dynamic and affected by growth conditions and stress (15,20–23). For example, the relatively compact nucleoid present in fast growing cells is altered by treatment with serine hydroxamate (SHX), which induces the stringent response (24) and inhibits replication initiation through artificial amino acid starvation. In terms of the biology of the *E. coli* nucleoid, the overall effect of the SHX-induced amino acid starvation is an expansion of the nucleoid and a change in transcription patterns (25,26). This suggests a relationship between transcription and the organization of the nucleoid (27). However, the mechanism(s) behind the re-structuring of the nucleoid in response to growth and stress is still largely unknown.

Another long standing question is when and how the nascent nucleoid that arises from DNA replication segregates during bacterial cell growth [reviewed in (1)]. In *E. coli*, the time required for the replication of the nucleoid is fixed at ∼40 min (28). To maintain a fast growth rate, cells growing in rich media must initiate multiple rounds of replication before each division. Consequently, a typical cell growing in rich media contains up to 16 origins of replication (29). Whether the nascent nucleoids segregate rapidly (30–32) or remain associated after replication, by a cohesion-dependent mechanism (i.e. the cohesion model) as seen in eukaryotes (33,34), remains unresolved.

Advances in chromosome conformation capture (3C)-related methodologies (35) enable the direct high-resolution detection of chromosome organization [e.g. (36–40)]. Recently, chromosome conformation capture carbon-copy (5C) was used to generate a global DNA:DNA contact map for *Caulobacter crescentus* synchronized swarmer cells (9). Here, we present a high-resolution analysis of the DNA:DNA interactions within *E. coli* nucleoids in rapidly growing and starved cell populations. Using genome conformation capture (GCC), we observe a clear relationship between DNA:DNA interactions, copy number and DNA replication. This suggests that nucleoids remain associated after replication, consistent with the cohesion model. Furthermore, SeqA-binding sites exhibit replication-dependent clustering, whereas binding sites for the major histone-like proteins (Fis, H-NS and IHF) did not. Finally, we observe a correlation between gene regulation and spatial clustering.

## MATERIALS AND METHODS

### Strains and growth conditions

For GCC analyses (36), *E. coli* strains (Supplementary Table S1) were recovered from −80°C on Luria Bertani (LB) agar (2%) plates (24 h, 37°C). LB medium (3 ml, Gibco) starter cultures were inoculated and grown (37°C, 220 rpm, 16 h). The optical density (OD_600_) of cultures was measured and used to inoculate LB test cultures to an OD_600_ of ∼0.02. The test cultures were grown (37°C, 220 rpm) until the OD_600_ reached ∼0.25, and the cells were harvested. For the SHX-treated samples, the cultures were treated with SHX (500 µg/ml, 30 min) before harvesting.

### Genome conformation capture

*E. coli* chromatin was prepared according to Rodley *et al.* (36) with minor modifications. In brief, 5 × 10^9^ formaldehyde cross-linked (1%) cells were lysed (Supplementary Materials and Methods) in the presence of protease inhibitor (Roche), and the chromatin was collected (21 500*g*, 20 min, 4°C). Chromatin was washed and suspended in chromatin digestion buffer (10 mM Tris–HCl, pH 8.0, 5 mM MgCl_2_ and 0.1% TritonX-100). Chromatin samples were digested with HhaI (100 U, New England Biolabs), diluted (∼20-fold) and ligated with T4 DNA ligase (20 U, Invitrogen). A ligation control was added to the digested chromatin (Supplementary Materials and Methods and Supplementary Table S2) before ligation. After ligation, cross-links, protein and RNA were removed. pUC19 plasmid was added as a sequencing control before three extractions with 1:1 phenol:chloroform. DNA was column purified (Zymo, DNA clean and concentratorTM^−^^5^ kit) according to the manufacturer’s instructions and eluted in milliQ H_2_O. Three micrograms of purified DNA was sent for paired-end sequencing (100 bp) at the ATC sequencing facility (Rockville, MD, USA) on an Illumina Hi-Seq.

### Genome conformation capture network assembly, effects of sample production and processing and bioinformatics analysis

To identify interacting DNA fragments from the paired-end sequence reads, network assembly was performed using the Topography suite v1.19 (41). GCC networks were constructed from 100-bp paired-end Illumina Genome Analyser sequence reads (Supplementary Materials and Methods). Except where indicated, bioinformatics and statistical analyses were performed on interactions identified by sequence reads that were uniquely mapped onto the reference genome and were above the cut-off value derived from the ligation control interactions (Supplementary Materials and Methods). A breakdown of the interactions present in the *E. coli* samples is provided in Supplementary Table S3. The effect of bar-coding, sequencing lane and biological replicates on the correlation between samples was quantified using the Cohen’s Kappa statistic, showing that these factors did not strongly affect sample correlations (Supplementary Materials and Methods). All bioinformatics analysis was performed using in house Perl and Python scripts (Supplementary Materials and Methods). Except where indicated, statistical analyses were performed in R (42).

### Genome copy number

Copy number was determined across the *E. coli* genome using control-free copy number and genotype caller (Control-FREEC) (43). The *E. coli* input sequences were in the SAM format, genome length was set at 4 639 675 bp, window size = 1000 and telocentromeric = 0. The GC profile was calculated and included.

### Transcription microarray

Briefly, similar to GCC, *E. coli* was grown in LB (Gibco, lot 817849) to an OD_600_ ∼0.2 and harvested directly, or first treated with SHX before RNA isolation. RNA was isolated using hot phenol and finally suspended in DEPC-treated water (Invitrogen). The cDNA libraries were constructed using a SuperScript Double-Stranded cDNA Synthesis Kit (Invitrogen) and sent to Roche-Nimblegen for microarray hybridization. Each experiment (exponential or SHX) is a pool of three biological replicates. A total of two technical replicates were performed per condition (exponential and SHX). Genes that were significantly up- or downregulated in SHX-treated compared with exponential samples were identified by calculating the log2 of the SHX/exponential ratio (Supplementary Materials and Methods and Supplementary Tables S4 and S5).

### MatS, SeqA, SlmA and NAP clustering analyses

NAP-binding sites were obtained from Grainger *et al.* (18). MatP-binding sites (MatS) were obtained from Mercier *et al.* (5). Regions for analysis were defined by taking a specified number of bases (50, 100 or 250 bp) either side of the peak binding position for NAPs or center of the MatP-binding site for MatS. For SeqA, the strongest 135 confirmed SeqA-binding sites were obtained from Sanchez-Romero *et al.* (44), and the 24 defined SlmA-binding sites were obtained from Cho *et al.* (45). To determine whether these regions could be found in a different interacting environment compared with what would be expected by random chance, the total number of interactions with each of the individual regions and the number of interactions that occurred between the regions of interest (i.e*.* clustering) was determined from our GCC interaction network. We then generated 1000 random data sets of the same number and length (bp) as the actual region data set using two methods: (i) randomly selecting a start position for each region and then making it the same length as the region for which the random coordinate was being generated [i.e. random spacing (RS)]; or (ii) randomly select the start position for the first region and then sequentially determining the start and end position of all the other regions in the set such that the linear distances between regions were maintained [i.e. conserved linear spacing (CLS)]. This ensured that the particular interaction frequencies we observed were not because of the linear arrangement of the regions around the circular genome. One thousand random data sets were generated for the RS and CLS methods, and the total interaction and clustering frequencies were calculated from our GCC interaction network. The frequency with which the total interaction and clustering frequency of the actual data was higher or lower than the random data sets was used to estimate significance.

### Interactions and clustering of genes that significantly change their expression level on SHX treatment

Genomic coordinates of genes that significantly change their expression level on treatment with SHX were obtained from http://regulondb.ccg.unam.mx/data/GeneProductSet.txt. The total number of interactions with each of the individual genes and the number of interactions that occurred between the genes of interest was determined as for MatS, SeqA, SlmA and NAP clustering, as described earlier in the text.

## RESULTS

**Figure 1.**
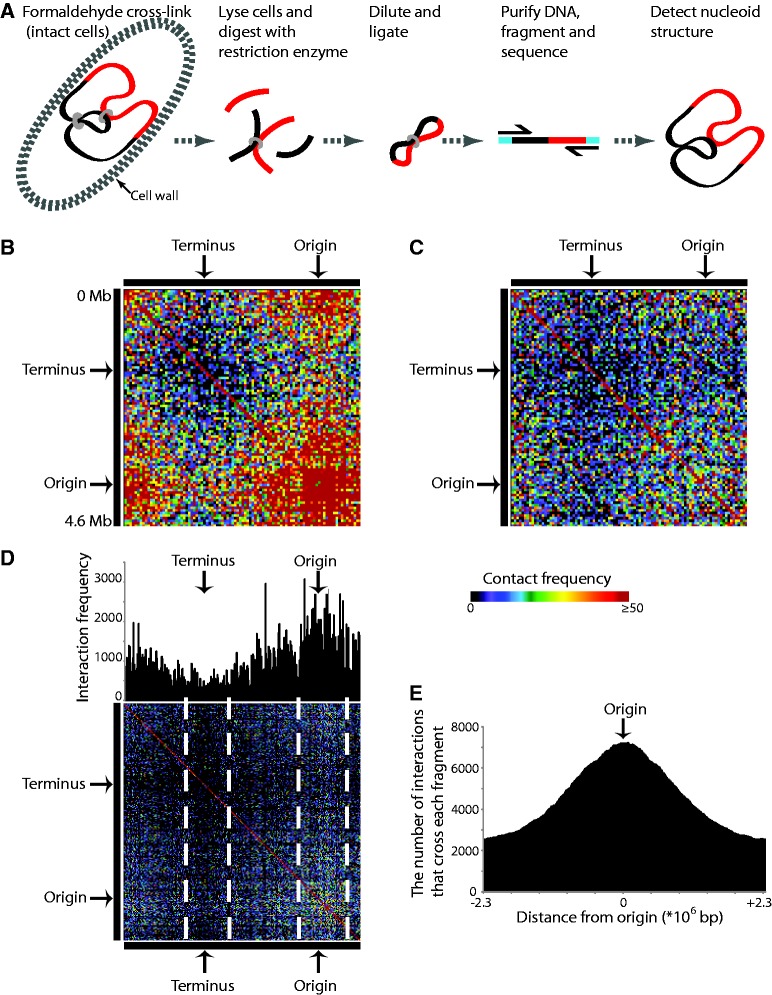
Ori and Ter domains are present within the *E. coli* nucleoid. (**A**) Schematic of the GCC procedure (36). Intact cells are cross-linked with formaldehyde before lysis, and the cross-linked nucleoids are isolated. The nucleoids are restriction digested, diluted and ligated to generate an interaction library. The interaction library is sequenced, after the addition of sequencing adapters (blue bars), and the network of interactions that define the nucleoid organization is determined. (**B**) Genome-wide contact matrix (50-kb bins) for exponentially growing *E. coli* nucleoids. The matrix highlights the Ori (high contact region) and Ter domains (low contact region). (**C**) Genome-wide contact matrix (50-kb bins) for nucleoids isolated from SHX-treated *E. coli*. The Ori and Ter domains remain visible. (**D**) Genome-wide contact matrix (20-kb bins) and bar graph for exponentially growing nucleoids highlighting regions of low interaction frequency (‘domain boundaries’) surrounding the Ori and Ter regions. (**E**) Frequency of exponential phase interactions that cross each restriction fragment plotted as a function of distance from the Ori (0). Fixed boundaries are not observed. The profile for the SHX-treated cells is not different (data not shown).

In GCC, the spatial organization of the nucleoid is captured by formaldehyde cross-linking within intact cells before cell lysis and the isolation of the nucleoid (Figure 1A). Once isolated, the nucleoid is digested, diluted and incubated with DNA ligase to enable the capture of spatially proximate but linearly separated loci (Figure 1A) (36). This produces an interaction library that can be sequenced to identify the network of chromosomal interactions occurring at the moment of cross-linking. GCC differs from current competing unbiased 3C technologies in that all DNA material is sequenced without the previous selection of DNA fragments containing ligation products. Therefore, there are no enrichment introduced biases, and DNA copy variation can be determined.

GCC relies on the intra-molecular ligation of cross-linked loci. However, inter-molecular ligation events resulting from random associations during the procedure can also occur, leading to false positives. To reduce the chances of isolating false positives, we (i) induce expansion of the nucleoid by isolation in a high-salt environment [a ‘high-salt nucleoid’ (2)], following cross-linking of the interacting loci; and (ii) added external ligation controls during GCC library preparations to empirically measure the background level of random inter-molecular ligation events. Thus, we determined a cut-off, for the minimum number of sequences representing any one interaction, above which interactions were deemed significant (Supplementary Materials and Methods). The following analyses were only performed on interactions that were above this significance threshold.

### Origin and terminus domains exist within the *E. coli* nucleoid

Chromosome interaction networks were determined for rapidly growing cells in rich medium harvested at early exponential phase and exponential cells treated with SHX (Figure 1B and C). The exponential phase chromosome interaction network (Figure 1B) is dominant in two regions: (i) a high frequency interaction domain surrounding the origin (Ori); and (ii) a low frequency interaction domain surrounding the terminus (Ter). These Ori and Ter domains are also present in the interaction network for the SHX-treated samples, although they are less pronounced (Figure 1C). Higher resolution (i.e. 20 kb) emphasizes that the exponential phase interaction network contains regions that have a demonstrably lower average interaction frequency than the adjacent Ori and Ter domains (Figure 1D). We attribute these reductions to the presence of non-fixed domain boundaries within the population. We predicted that these boundaries would reduce interactions between domains, and that this would be manifested as a reduction in the interactions that cross the boundary regions. However, despite the obvious Ori preference, there is no sharp reduction in the numbers of interactions that cross our apparent domain boundaries (Figure 1E). Despite the diffuse boundaries for the Ori and Ter domains, we observe several noticeable reductions in the interaction frequency at various locations in the chromosome that could represent additional domain boundaries.

### Interactions within the Ori and Ter regions are linked to replication

**Figure 2.**
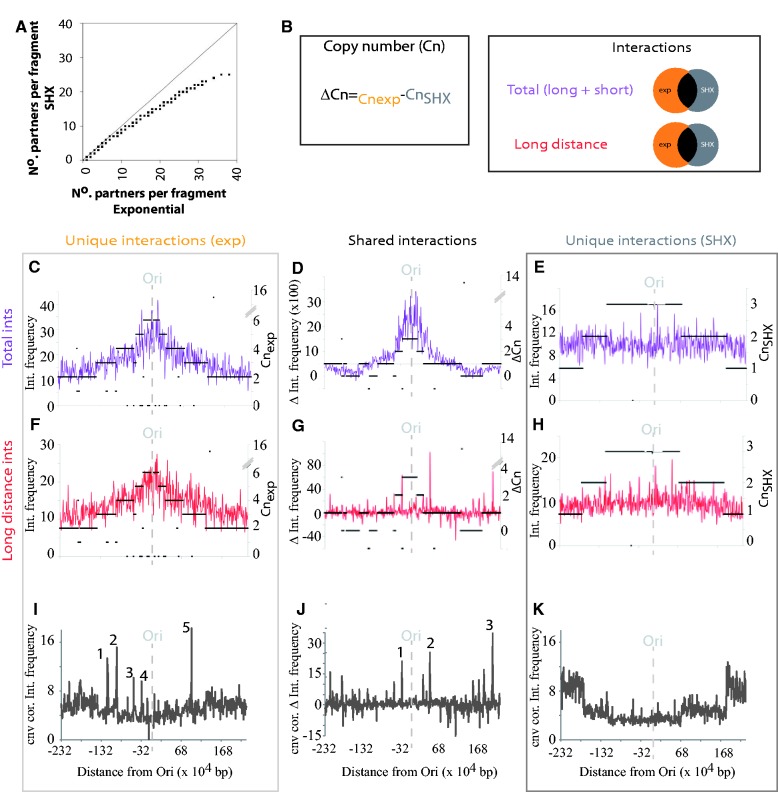
Origin proximal interactions are more frequently detected. (**A**) Fragments that interact have more partners in the exponential nucleoids as opposed to SHX-treated nucleoids. The 45° line shows the expected pattern if the number of partners for each fragment is equal in both conditions. (**B**) Schematic of the copy number and interaction comparisons that were performed. Comparisons between interaction frequency and copy number: (**C–E**), total observed interactions; (**F–H**), long distance (>800 bp) interactions. (C) Interactions that are specific to exponential phase growth correlated with copy number. (D) Differences in frequency for shared interactions between exponentially growing and SHX-treated *E. coli* cells indicate a correlation with copy number. (E) Interactions that were specific to SHX-treated cells are copy number independent. (F) Exponential phase-specific long distance interactions correlated with copy number. (G) Removal of short distance interactions (≤800 bp) removed the copy number dependence of the shared interactions. (H) SHX-specific interactions were independent of copy number. (**I**) Correction of exponential-specific long distance interactions identifies five peaks (I1–5) of increased interactions at positions (I1) 2 753 883–2 773 883, (I2) 2 983 883–3 003 883, (I3) 3 413 883–3 423 883, (I4) 3 613 883–3 623 883 and (I5) 224 208–234 208 bp. (**J**) Correction of shared long distance interactions identifies three peaks (J1–3) of increased interactions at positions (J1) 3 643 883–3 653 883, (J2) 4 383 883–4 393 883 and (J3) 1 404 208–1 414 208 bp. (**K**) Correction of SHX-specific long distance interactions for copy number identifies a decrease in the relative frequency of interactions at the origin compared with the terminus. Interactions were tallied for 10 000-bp bins and corrected for the number of fragments per bin. Vertical, gray broken lines denote the position of the origin of replication. Copy number is depicted by black horizontal bars.

Comparisons of the chromosome networks from the exponential and SHX-treated cells identified similar levels of self and adjacent interactions (Supplementary Table S3). However, SHX treatment results in fewer long distance interactions (between 800 bp and half the length of the genome, respectively; Supplementary Figure S1A), shorter loop lengths (Supplementary Figure S1B) and reduced numbers of partners per fragment (Figure 2A and Supplementary Figure S2) when compared with the exponential network. These observations are consistent with SHX, decreasing the overall compaction of the nucleoid (21–23).

The high frequency of replication initiation in rapidly growing cells leads to an enrichment of origin-proximal loci, which could explain the pronounced increase in the number of partners observed in this region in exponentially growing cells (Supplementary Figure S2A). By contrast, treatment with SHX reduces this bias (Supplementary Figure S2B). These results are consistent with the inhibition of replication initiation after SHX treatment leading to a reduction in the Ori:Ter copy number ratio (46) or structural alterations within the origin domain.

To investigate whether interaction frequencies are affected by differences in copy number across the bacterial chromosome because of DNA replication, we compared interaction patterns and copy number before and after SHX treatment. Interactions were grouped according to the linear distance between the interacting loci and occurrence in the different environmental conditions (Figure 2B and Supplementary Table S3). The distribution of interaction strength and copy number relative to the origin was determined (Figure 2C–K). Exponential phase-specific and shared short distance interactions correlate with copy number (Figure 2C, D and F). By contrast, SHX-specific or shared long distance interactions do not correlate with copy number (Figure 2E, G and H). Critically, the ratio of Ori to Ter regions within both the exponential and SHX conditions remains at 3:1 (compare copy number Figure 2C and E). Thus, the observed decrease in the frequency of the interactions within the origin domain (compare Figure 1B and C) is either because of a decrease in the absolute number of origin sequences or because of a structural alteration (e.g. expansion) of the Ori domain.

Correcting the frequency of long distance interactions by copy number, a feature of GCC, indicates that most genomic regions interact with similar frequencies within the exponential-specific and shared interaction sets (i.e. interactions that occur in both the exponential and SHX conditions; Figure 2I and J). However, there are several notable deviations from this trend (labeled peaks within Figure 2I and J). The observed deviations are due to interactions involving multiple fragments within each of the 10 000-bp segments that are plotted (Figure 2I and J). By contrast, copy number correction of the long distance SHX-specific interactions identifies an increase in the interaction frequency within the Ter domain. The remainder of the genome shows relatively even and low interaction frequencies within the SHX-specific interaction set (Figure 2K).

### Clustering of MatP- and SeqA-binding sites links nucleoid structure and replication

To further investigate the link between replication and nucleoid organization, we determined the clustering and interaction properties of loci containing characterized protein-binding sites for the MatP, SlmA and SeqA proteins.

**Figure 3.**
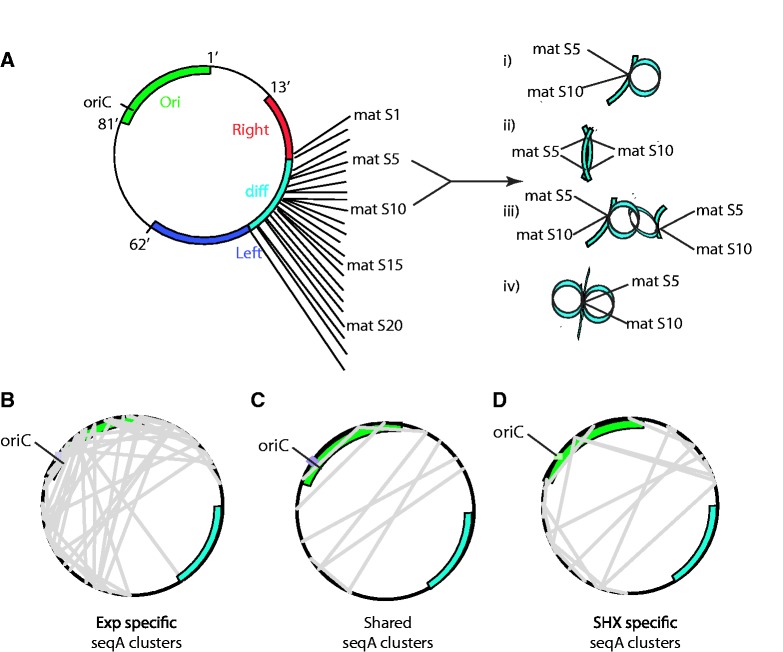
Binding sites for NAPs MatP and SeqA exhibit differing degrees of spatial clustering within the exponential and SHX-treated *E. coli* nucleoids. (**A**) Regions that centered on matS-binding sites [±50 bp; (5)] show significantly increased clustering in the exponential condition, despite having interaction levels that were no different from random (Supplementary Table S6). MatS site clustering is confined to two matS sites: matS5 and matS10 and may result from (A i) intra-chromosome interactions, or (A ii–iv) inter-chromosomal interactions. Critically, this clustering is not observed in the SHX-treated nucleoid. (**B**) Exponential-specific spatial clustering of SeqA-binding sites was concentrated around the origin. (**C**) Spatial clusters of SeqA-binding sites that were shared between conditions tended to occur between the left and right replichores. (**D**) SHX-specific interactions involved fewer SeqA-binding sites and tended to be toward the terminus (Supplementary Table S7).

MatP is a protein that binds to matS sites and organizes the Ter macrodomain (5). Analyses of matS loci identify significantly (*P* < 0.008) high clustering (i.e. inter-matS loci interactions) within the exponentially growing cells (Supplementary Table S6). In contrast, clustering of matS sites was not detected in the SHX-treated cells. The clustering in the exponentially growing condition was attributed to a single specific interaction between matS10 and matS5 (Figure 3A). This interaction must result from intra- or inter-Ter associations of these matS sites (Figure 3A i–iv).

The finding that SeqA binds as a dimer, which multimerizes to form a left-handed filament [reviewed in (47)], suggests that this protein may link spatially separated binding sites. Clustering of the 135 strongest confirmed SeqA-binding sites present within exponentially growing *E. coli* (44) was significantly higher than the random set (*P* < 0.05) (Supplementary Table S7). Moreover, these sites are significantly more prone to interact with other loci than random sites (*P* < 0.05; Supplementary Table S7). Visualizing the positions of the SeqA–SeqA interactions that formed within the *E. coli* genome showed that they tend to occur toward, and involve, the Ori domain in exponential cells (Figure 3B and C). SeqA interactions that are shared between exponential and SHX-treated nuclei predominantly link the left and right replichores (Figure 3C). By contrast, cells treated with SHX have a reduction in clusters involving SeqA sites surrounding the Ori domain and more inter-replichore interactions toward the terminal domain (Figure 3C and D). This is consistent with the progression of active replication forks that were initiated before SHX treatment.

SlmA binds at 24 defined sites within the genome (45) and acts to prevent FtsZ polymerization and premature cell division before complete chromosome replication. Analyses of the clustering and interaction profiles of *E. coli* SlmA sites demonstrated that clustering of these sites was not different from that observed for randomly selected sites (Supplementary Table S8). However, SlmA sites did exhibit a significantly increased propensity to interact with other genomic loci (*P* < 0.05) compared with randomly spaced elements for both exponential and SHX-treated cells (Supplementary Table S8). The significant increase in interaction frequency was lost when comparisons were made with random sets that have conserved linear spacing (Supplementary Table S8). Note that the differences observed in significance when the test data set was compared with randomly generated data sets (i.e. RS or CLS) confirm that the linear spacing of *E. coli* loci is important. Whether this is an effect or cause of spatial organization remains to be determined.

### Intra- or inter-NAP–binding site clustering does not contribute to the global organization of the *E. coli* nucleoid

We investigated the clustering and interaction properties of H-NS-, IHF- and Fis-binding sites, which are not enriched in any particular macrodomain. There is no detectable clustering for the 200-bp regions surrounding the Fis-, H-NS- and IHF-binding sites in either the exponential or SHX-treated nucleoids (Table 1). Moreover, the classical NAP-binding sites have depleted levels of interactions in exponentially growing *E. coli* cells (Table 1). These results can be explained by restrictions in the flexibility of the DNA (and, hence, reduced ligation efficiencies) because of the binding of the NAP. However, increasing the length of the region surrounding the binding site has no effect on the clustering (data not shown). Additionally, we do not observe intra-NAP–binding site clustering (Table 1), consistent with the temporal isolation of the expression of these NAPs (48).

**Table 1.** H-NS, IHF and Fis sites do not exhibit spatial clustering

	Interaction set	Genic regions		Non-genic regions		Clustering		Interactions	
		Clustering RS	Interactions RS	Clustering RS	Interactions RS	RS	CLS	RS	CLS
NAP^a^									
Fis	Exp	ND	Low**	ND	Low**				
	SHX	ND	NC	ND	Low**				
H-NS	Exp	ND	Low**	ND	Low**				
	SHX	ND	Low*	ND	Low**				
IHF	Exp	ND	Low*	ND	Low**				
	SHX	ND	NC	ND	Low**				
NAP (500 bp)^b^									
Fis, H-NS, IHF coding	Exp					NC	NC	Low**	Low**
	SHX					Low*	NC	Low***	Low***
Fis, H-NS, IHF non-coding	Exp					Low**	Low*	Low**	Low**
	SHX					Low**	NC	Low**	Low**

^a^The summed interaction or clustering strength for each condition was compared with 1000 random data sets with RS or CLS between each element (see ‘Materials and Methods’ section). Clustering of characterized H-NS-, IHF- and Fis-binding sites [±100 bp; (18)] located within coding and non-coding sequences was not detected. H-NS-, IHF- and Fis-binding sites had significantly lower interaction frequencies than random. However, treatment with SHX altered the interaction frequencies of genic H-NS and Fis sites such that they were no longer different from random. CLS results were the same as those observed for the RS. Short distance (<800 bp) and self-interactions were excluded from these analyses.

^b^Clustering of loci that contained one or more characterized H-NS, IHF and Fis sites (±500 bp) was no different to random (coding loci) or lower than random (non-coding loci), confirming that these elements do not cluster either individually or collectively.

*P < 0.085; **P < 0.001; ***P < 0.05.

NC, no change; ND, not detected.

### Genes up- or downregulated after SHX treatment exist in different spatial environments, confirming functional compartmentalization of the nucleoid

**Figure 4.**
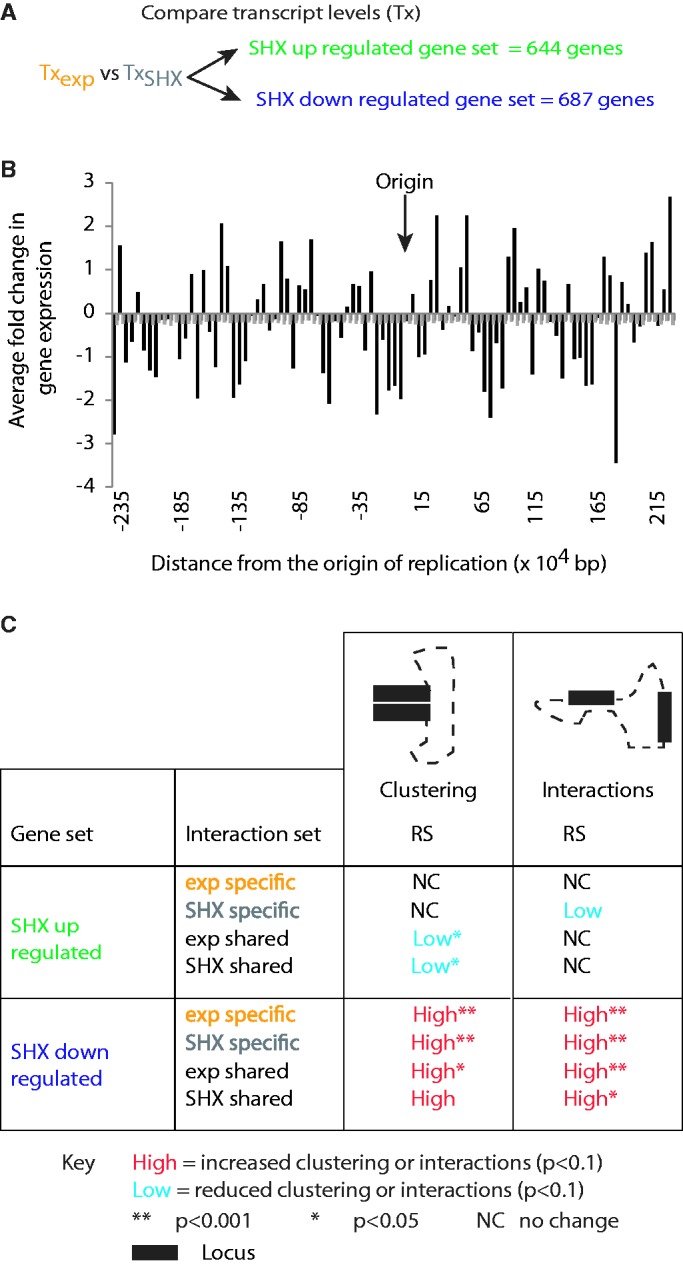
Annotated genes with transcripts that were up- (644 genes) or down- (687 genes) regulated after SHX treatment existed in different spatial environments. (**A**) Genes that changed transcript level (Tx) after treatment with SHX were identified. (**B**) Analyses of positions of the up- and downregulated genes across the *E. coli* genome identify non-random clustering within the linear sequence. Average expression levels were calculated for 50-kb bins. Grey bars indicate the average expression across 50-kb bins within a thousand randomized genomes. Autocorrelation analyses on the distribution of gene expression data across the genome demonstrated a strong predictive relationship up to 32 genes away (ACF: >0.83). (**C**) Clustering and interaction patterns for up- or downregulated genes demonstrate that up- and downregulated genes occupy specific spatial environments. The amount of clustering within the up- or downregulated gene sets, and between the up- or downregulated genes and other loci, was compared with 1000 randomly generated sets. One thousand random sets of equivalent size (number and length) to the up- or downregulated sets were generated such that they (i) randomized the spacing between elements (RS) or (ii) conserved the linear spacing between the elements (CLS) involved in the interactions. Clustering and interaction counts were determined individually for the condition specific and shared data sets. Clustering and interaction data are shown for both exponential (exp) and SHX shared interaction sets because despite the interaction being shared, the clustering or interaction frequency was specific for each condition. There were no significant differences for comparisons with either the RS or CLS random sets. These analyses were performed on long distance interactions only.

Eukaryotic studies have identified a non-random distribution of gene expression associated with the presence of spatially distinct environments that promote or inhibit nuclear functions [e.g. (49–51)]. Similarly, we observe that *E. coli* genes whose transcript levels increased or decreased in response to SHX treatment are overrepresented in some gene ontology terms (Supplementary Table S5) and are non-randomly distributed across the linear genome (Figure 4A and B) in a manner that does not correlate with GC content (Supplementary Figure S3A). There is no correlation between transcript level and interaction frequency at the level of specific restriction fragments (Supplementary Figure S3B and C). However, the SHX downregulated genes have high average transcript (*P* < 0.001; Supplementary Table S9), clustering and interaction (Figure 3C) levels in exponential phase cells. These results suggest that genes that are highly expressed in exponential phase and downregulated after SHX treatment are not only linearly but also highly spatially clustered. In conjunction with microscopic observations of large RNA polymerase clusters (foci) within exponentially growing *E. coli* cells (21), our results support the hypothesis that the highly expressed exponential phase genes are associated with transcription foci. Despite this, genes downregulated in response to SHX treatment (*P* < 0.001; Supplementary Table S9) remained highly clustered (Figure 4C). Similarly, upregulated genes within lowly clustered regions do not increase their clustering on activation (Figure 4C). As such, the maintenance of the clustering is independent of transcript levels and *ipso facto* transcription.

## DISCUSSION

The *E. coli* nucleoid has a complex structure that emerges from the sum of the cellular processes that occur within the bacterial cell. We identified two macrodomains within the *E. coli* chromosome interaction networks corresponding to the Ori and Ter domains that have been previously identified (5,7,8,12,13,52). However, the two remaining macrodomains [Left (L), Right (R)] and the two non-structured domains (NS) are not obvious within our data. Moreover, we did not identify hard boundaries surrounding either the Ori or Ter domain, consistent with earlier predictions (7,12). It remains possible that the L, R and NS domains and the domain boundaries were obscured because of the use of an unsynchronized population of cells. Alternatively, the formation of the macrodomains and the previously observed reductions in inter-domain recombination rates (12) could be achieved by a combination of mechanisms of which physical segregation is only one component. This explanation is supported by the observation that a low level of connectivity remains between the Ter and Ori domains. Critically, this connectivity occurs at levels above those observed for random inter-molecular ligation under our experimental conditions and indicates that although these domains are largely separated, there is some inter-domain mixing during the cell cycle. This is consistent with the observation that recombination rates between λ att sites are reduced but not completely abolished between these domains (12).

The chromosome interaction networks we identified within both exponential and SHX-treated *E. coli* cells contain variable numbers of short and long distance loops. The observation that the number of long distance interactions (long distance loops) reduced after treatment with SHX can be interpreted as indicating that the nucleoid expands under this condition, consistent with microscopic observations (21,22,53). Either the observed expansion is specific and directed as part of the stress response or it is a non-specific consequence of SHX acting on the factors that mediate the interactions (e.g. rapid protein turn over with no replacement). The exact reasons for the loss of interactions remain to be determined. However, the fact that SHX-specific interactions form indicates a directed alteration in nucleoid organization.

### Is the *E. coli* nucleoid shaped as a sausage or rosette?

**Figure 5.**
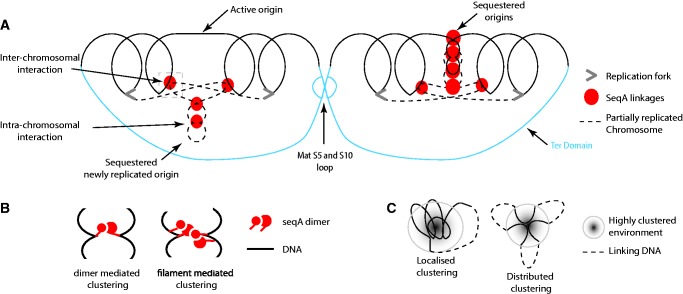
Spatial model of exponential phase nucleoid organization in *E. coli*. (**A**) The exponential phase *E. coli* nucleoid is organized into high interacting domains by nucleoid-associated factors including, but not limited to, SeqA and MatP. SeqA promotes the intra- and inter-chromosomal clustering of hemimethylated GATC sites to sequester recently replicated origins and contribute to chromosome segregation. Newly replicated origins can be sequestered individually (left) or through interactions between the recently replicated origins (right). The matS5–10 loop is hypothesized to form between chromosomes that have almost completed replication. The model illustrates some of the major findings in the study, but for simplicity, overlap between replichores has not been included in this cartoon. Similarly, only one replication process has been illustrated on each chromosome. Moreover, as our data are drawn from an unsynchronized population, and we do not have data on the relative positions of the different elements through the cell cycle, we have not attempted to represent the dynamic nature of the positioning of the different elements throughout the cell cycle. (**B**) SeqA can mediate interactions within or between chromosomes as either a dimer or filament. (**C**) Highly clustered regions form as a result of localized and distributed clustering within and/or between the replicated chromosomes.

The presence of short and long distance loops within both networks points to the *E. coli* genome folding into a series of DNA loops connected to a central node (i.e. a rosette). This interpretation agrees with electron microscope observations of isolated nucleoids [reviewed in (2)]. However, our observation that the Ter region has few contacts with itself (i.e. is extended in nature) and is less well connected to the remainder of the genome is consistent with previous observations made by David Sherratt’s group (4,54). Therefore, despite differences in growth rate between the studies (4), our data also support the hypothesis that the *E. coli* chromosome is organized as a sausage in which the bulk of the chromosome is organized into a compacted rod that is circularized by the Ter domain [Figure 5A (4,54)]. The apparent dichotomy of these interpretations is reconcilable through the realization that the isolation of a sausage-shaped genome during preparation for electron microscopy would result in the appearance of a rosette. Thus, the sausage model is a variation of the rosette model where the rosette is flattened through confinement or as a result of the biological processes within the live cell.

### Replication contributes to nucleoid organization through SeqA

The SeqA and SlmA proteins are implicated in the regulation of replication and chromosome separation [reviewed in (16)]. Our results indicate that SlmA-binding sites do not cluster as part of nucleoid occlusion during replication initiation or extension. Therefore, the dimerization necessary to activate SlmA occurs at a single or linearly-adjacent binding site(s) but does not result from spatial associations of distant SlmA sites. Consistent with the supposition by Dame *et al.* (16), the low levels of SlmA clustering observed indicate that any contribution that SlmA-FtsZ makes to nucleoid structure must be facilitated by tethering to an external framework [e.g. shortened preformed FtsZ polymers (45), or non-functional protofilaments (55)] or the cell membrane.

By contrast, the replication-dependent nature and distribution of the exponential phase SeqA-mediated long distance interactions provides support for a role for SeqA clustering in the formation of an intra- and/or inter-chromosomal structure (Figure 5A and B). This is particularly true for SeqA interactions that form over the origin of replication and could function to sequester newly replicated origins and delay chromosome separation [(56–58), reviewed in (16,47)]. As such, the SHX-dependent loss of the long distance interactions is predicted if replication and segregation occur consecutively (29). Thus, the loss of SeqA-mediated interactions within the SHX-treated nucleoid reflects an underlying spatial segregation of the replicated chromosome regions (46). The predominance of SeqA clusters between loci that are approximately equidistant from the Ori within the SHX–specific, and shared interaction data sets represent links between the hemimethylated GATC sites trailing the replisome. We interpret the distinct subset of inter-replichore SeqA clusters as indicating that the DNA polymerases are pausing at specific genomic sites within the cell populations. Finally, there is no correlation between alterations to transcript levels and SeqA clustering (data not shown); therefore, SeqA clustering is independent of transcription. Collectively, these results support a strong linkage between replication and nucleoid organization (4).

For ease of visualization, the chromosomal interactions that we identified are presented as intra-chromosomal connections (Figure 1). This form of presentation is problematic, as the proximity-based ligation data are probabilistic and represent a population average from unsynchronized cells (59). As such, it is impossible to determine which combinations of interactions occur within a single nucleoid. Second, although the sequences we obtain as part of the GCC protocol identify the interacting loci, they do not provide information on whether the interactions occur within or between the chromosome(s). This is an important consideration when investigating nucleoid structure in exponential phase bacterial cells that contain and segregate partially replicated chromosomes (3). Therefore, it is possible that the formation of long distance SeqA-dependent and -independent interactions can be facilitated by overlaps between the replichore arms that result from the chromosome alignment [i.e. inter-chromosomal (Figure 5A, right)]. Interestingly, such a system may contribute to gene dosage control, as well as the control of chromosome segregation. However, it remains possible that interactions also occur within a chromosome [i.e. intra-chromosomal (Figure 5A, left)]. Future work should determine the contribution of inter- and intra-chromosomal interactions to the structure of the nucleoid in exponentially growing *E. coli* with a view to understand how structure contributes to gene dosage control in this organism.

### What role does the matS5–10 loop play in nucleoid organization?

MatS sites have a role in defining the Ter domain (5,8). *In vivo* experiments indicate that the definition of the Ter domain and condensation of this region are separable events with the condensation dependent on the presence of the MatP C-terminal coiled-coil domain, which is responsible for tetramerization and looping (60). We found that the matS5 and matS10 sites form a specific loop that surrounds the TerA site (1 339 796–1 339 791 bp) and is located away from the dif site (1 589 000 bp) toward the Ori on the right replichore. Note that matS5 is one of two matS sites (the other being matS21) that do not show *in vivo* MatP binding in an *E. coli* K12 derivative of MG1655 (5). The question thus arises as to what contribution the matS5–10 interaction makes to the Ter domain structure and function. It is possible that the matS5–10 loop explains observations of a spatially separable condensed region within the center of the Ter linker domain (4). Furthermore, the absence of detectable matS clustering between the other matS loci raises the possibility of differentiation in the functions of the matS sites. However, further experiments are required to confirm these hypotheses and identify how or if MatP contributes to the formation of the matS5–10 loop.

### Do ‘histone-like’ NAPs play a role in global nucleoid structure?

The spatial clustering of NAP (i.e. H-NS, Fis and IHF) DNA-binding sites is not significant within the gross spatial organization of the *E. coli* nucleoid we identified. Rather our results are consistent with the hypothesis that H-NS, IHF and Fis contribute to compaction through localized structuring [reviewed in (61)], gene regulation or the formation of large protein heterocomplexes [reviewed in (62)]. These results are in contrast to those of Wang *et al.* 2011 (14), who identified H-NS clustering within the *E. coli* nucleoid using microscopic and proximity-ligation–based measurements in slow-growing early log phase cells. This apparent discrepancy may be due to the significant increase in resolution afforded by the use of the HhaI enzyme in our study. This conclusion is supported by our identification of interactions linking HhaI restriction fragments from within the larger EcoRI restriction fragments that were previously characterized as demonstrating an H-NS–dependent association [Supplementary Figure S4 (14)]. Therefore, we propose that the previously recognized relationship between ligation efficiency and the presence/absence of *h-ns* mutants (14) was likely due to a combination of a global reorganization of localized genome structure (63) and epistatic effects resulting from H-NS–dependent transcriptional changes.

### Do transcription foci have a role in nucleoid organization?

The observed organization of highly transcribed genes into clustered spatial environments is consistent with the hypothesis that some clustering is occurring around transcription foci [e.g. (64)]. Similarly, the copy-number independent long distance interactions may reflect sequence-driven intra-chromosomal nucleoid folding for the coordination of transcription through enhancer-like interactions consistent with previous observations in bacteria (14,65,66) and eukaryotes [e.g. (67–69)]. The existence of these prokaryotic transcription foci is supported by microscopic observations of RNA polymerase foci within *E. coli* cells (20,21). The fact that similar clustering was observed in *Pseudomonas aeruginosa* (data not shown) and among highly transcribed genes in *Schizosaccharomyces pombe* (40) implies that the clustering of highly transcribed genes may be a ubiquitous feature of the control of gene expression.

It is likely that the linear gene clusters (Figure 4A) form into combinations of localized and distributed spatial clusters (Figure 5C). Given that RNA polymerase is redistributed after SHX treatment (21,22), decreases in the number of long distance interactions (i.e. reductions in the extent of distributed clustering), we observed following stress induction could be interpreted as indicating that RNA polymerase mediates some interactions. However, the identification of a core interaction pattern that is conserved within the *E. coli* nucleoid after SHX treatment indicates that at least some of these interactions are stable to a significant redistribution of RNA polymerase. This result agrees with eukaryotic studies that demonstrate long distance interactions are insensitive to inhibition of ongoing RNA polymerase transcription (70). Furthermore, the high levels of clustering and interactions observed at genes that were highly expressed in the exponential phase and subsequently downregulated by SHX treatment indicates that the localized clustering—but not necessarily the identity of the partners—is stable. However, it remains possible that transcription-associated interactions respond slowly to environmental change, allowing for short term fluctuations in environmental conditions without the requirement for major rearrangement of genome organization. This forms an epigenetic memory that is capable of being inherited (71) similar to that observed in yeast (72–76).

### Does a nucleolus-like structure form within the *E. coli* nucleoid?

It has been proposed that the formation of transcription factories that include the ribosomal RNA genes and ribosomal protein encoding loci could induce the compaction of the nucleoid through the formation of a nucleolus-like structure (23,77,78). However, we found no evidence that the nucleoid structure promotes the clustering of ribosomal RNA genes and ribosomal protein encoding loci (data not shown). This may be due to technical limitations in the analysis of repetitive loci that cannot be unambiguously positioned onto the reference genome. Alternatively, it may be due to the very high levels of transcriptional activity at these loci interfering with the cross-linking and ligation steps during the preparation of our chromosome interaction libraries. *In silico* modeling of the nucleoid that incorporates biophysical parameters and interaction frequencies [similar to (9,79)] may resolve this issue.

### Epistatic interactions and the chromosome interaction network

The bacterial cell is a complex structured entity in which each part exists ‘for and by means of the whole’ (80). As such nucleoid structure is an integral—inseparable—part of the cells response to environmental challenge. Moreover, the contribution of any one gene to the bacterial phenotype relies on its relationship with other genes on levels that include regulation, transcription, translation, complex formation and function. Therefore, it is likely that the interaction network we have determined contains information on epistatic relationships between multiple genes that occur at the regulatory, transcriptional and translational levels because of the co-dependence of these processes in *E. coli*. Future work should interrogate prokaryotic interaction networks for evidence of epistatic relationships and must address the mechanism(s) governing the organization of global structure.

## CONCLUSION

The detection of both long and short distance interactions within the *E. coli* nucleoid is consistent with empirical measures and modeling, which indicated that intra-nucleoid interactions play a dominant role in shaping the *E. coli* nucleoid (11). However, the long distance interactions did not consistently involve loci located equidistant from the Ori on opposite replichores; therefore, it is unlikely that the *E. coli* nucleoid is preferentially structured as ellipsoids as observed in *C. crescentus* (9). Rather our study indicates that the chromosome(s) within exponentially fast-growing *E. coli* cells are structured by interactions that are linked to the ongoing replication and transcription processes within the cell. The specificity of the observed interactions identifies spatial organization as a significant factor in bacterial gene regulation and indicates that the spatial clustering of highly regulated genes is a ubiquitous feature of gene regulation.

## ACCESSION NUMBERS

The GCC data has been banked with Gene expression omnibus (GSE40603). Expression data has been deposited GSE40304.

## SUPPLEMENTARY DATA

Supplementary Data are available at NAR Online: Supplementary Tables 1–10, Supplementary Figures 1–6 and Supplementary Materials and Methods.

## FUNDING

Intramural Research Program of the National Institutes of Health, National Cancer Institute, Center for Cancer Research (to C.C. and J.D.); the Marsden Fund (to J.M.O.S.); Massey University research fund (to J.M.O.S and B.H.A.R.); Massey University scholarship (to R.S.G.). Funding for open access charge: The Liggins Institute Auckland University.

*Conflict of interest statement.* None declared.

## Supplementary Material

Supplementary Data
