# Supplementary material for: Genome conformation capture reveals that the Escherichia coli chromosome is organized by replication and transcription
Source: Nucleic Acids Res. 2013 Apr 30;41(12):6058–71. doi: 10.1093/nar/gkt325 (PMC3695519; doi:10.1093/nar/gkt325)
Supplement: Supplementary Data [file supp_41_12_6058__index.html]

Genome conformation capture reveals that the Escherichia coli chromosome is organized by replication and transcription — Genome conformation capture reveals that the Escherichia coli chromosome is organized by replication and transcription — Supplementary Data 

# Genome conformation capture reveals that the *Escherichia coli* chromosome is organized by replication and transcription

## Supplementary Data

files

**Files in this Data Supplement:**

- Supplementary Data - pdf file
