# Supplementary material for: Genome conformation capture reveals that the Escherichia coli chromosome is organized by replication and transcription
Source: Nucleic Acids Res. 2013 Apr 30;41(12):6058–71. doi: 10.1093/nar/gkt325 (PMC3695519; doi:10.1093/nar/gkt325)
Supplement: Supplementary Data [file supp_gkt325_nar-00652-h-2013-File008.pdf]

Supplementary Materials and Methods

Genome Conformation Capture (GCC)

E. coli chromatin was prepared according to (1) with the following modifications. A total of $5*10^9$ cells were cross-linked with formaldehyde (1% final v/v, 20min, RT) and then quenched with glycine (125mM final, 10min). Cells were collected by centrifugation (4000rpm, 15min, 4°C), washed twice (1% PBS, 1% TritonX-100, 5ml/50ml culture) and pelleted (4000rpm, 15min, 4°C). Cell pellets were suspended in 800µl of B1 lysis buffer (10mM Tris pH 8.0, 50mM NaCl, 10mM EDTA, 20% (w/v) sucrose, 1mg/ml lysozyme) and incubated (37°C, 30min). 800µl of B2 lysis buffer (200mM Tris pH 8.0, 600mM NaCl, 4% TritonX-100, 1 protease inhibitor tablet (Roche) per 10ml of buffer added just before use) was gently added, mixed by inversion 3-4 times and incubated (37°C, 10min). The cell lysate was centrifuged (21,500g, 20min, 4°C) and the supernatant decanted. The chromatin was washed once with 1ml of chromatin digestion buffer (10mM Tris-HCl pH 8.0, 5mM MgCl2, 0.1% TritonX-100) by inverting the tube 3-4 times and centrifuged (21,500g, 20min, 4°C). The supernatant was decanted and the chromatin pellet was suspended in 500µl chromatin digestion buffer. Chromatin samples were aliquoted into 10 sets of $5*10^8$ cells. Samples were digested with HhaI (100U, New England Biolabs). A ligation control was added to the digestion chromatin (see below and Table S2), the samples were diluted (~20-fold) and ligated with T4 DNA ligase (20U, Invitrogen). Following ligation, cross-links were removed in the presence of proteinase K (0.45U, Fermentas). RNA was removed and pUC19 plasmid (27.4pg/2ml) was added as a sequencing control prior to three extractions with 1:1 Phenol:Chloroform. DNA was column purified (Zymo, DNA clean and concentratorTM^{-5} kit) according to the manufacturer's instructions and eluted in milliQ H_2O before combining for sequencing. 3µg of purified DNA was sent for paired-end sequencing (100 bp) at the ATC sequencing facility (Rockville, MD, USA) on an Illumina Hi-Seq.

Production of external ligation controls for GCC library preparation

External ligation controls were produced by PCR amplification of short regions from the Lambda phage genome and the pRS426 plasmid. Primers (Table S2 were designed to include an HhaI site at one end of the final product. PCR products were purified using a PCR purification kit (Qiagen), digested with HhaI (4U, 37°C, 2h) and purified again. Purified, digested PCR products were introduced into the GCC samples at a 1:1 ratio with the number of genomes prior to the ligation step during GCC preparation. The pRS426 fragment was introduced into the exponential phase (LB grown) samples and resulted in 220 separate ligation events with HhaI restriction fragments on the genome. The Lambda phage fragment was introduced into the SHX treated samples and resulted in 2 ligation events with HhaI fragments on the genome.

Network Assembly

Network assembly was performed using the Topography suite v1.19. GCC networks were constructed from 100 bp paired-end Illumina Genome Analyser sequence reads. Topography uses the SOAP algorithm (2) to position PE tags and single ends which contain a HhaI restriction enzyme site onto the *E. coli* (NC_000913) reference genome. The reference genome also contained the pUC19 (SYNPUC19CV) sequence and the sequences of the pRS426 plasmid and Lambda phage ligation controls (Table S2). No mismatches or unassigned bases (N) were allowed during positioning.

Except where indicated, bioinformatics and statistical analyses were performed on interactions in which the sequence reads were able to be mapped uniquely onto the reference genome and were above the FDR cut-off value (see below). All bioinformatics analysis was performed using in house Perl scripts. Breakdowns of the interactions present in the *E. coli* samples are provided in Tables S3.

The use of paired-end (PE) sequencing reads means that the HhaI restriction enzyme site does not have to be present in either of the sequences to detect an interaction. This however results in all sequences effectively resulting in an interaction. Therefore, to insure that we could detect the same interactions using single ended reads, which necessitates that there is a HhaI restriction enzyme site detected in the sequence, we reconstructed part of the network using single-end (SE) reads and compared the interactions we identified to those detected in the PE analysis. Sequences from one of the two *E. coli* exponential phase biological replicates were used for SE network assembly as above. The number of interactions detected in this data set was compared to the interactions detected in the PE networks for the combined biological replicates. This analysis demonstrated that 92% of the interactions detected in the PE analysis were detected in the SE analysis of one of the two biological replicates for the exponentially growing cells. Therefore, the interactions detected by PE sequencing

are real and not just an artefact of the PE analysis.

Collector's curve

Collector's curves were generated using the total interaction data set from exponential growing and SHX treated *E. coli* cells, including the internal ligation controls. Specified fractions of the total interactions (e.g. 10%, 20%,…) were randomly sampled 100 times. For each random data set, the fragments that had an interaction frequency strictly greater than the number of interactions detected between *E. coli* genomic loci and the internal ligation controls were considered significant. The significant interactions were compared to the significant interactions present in the original data file containing only significant non-adjacent interactions. The number of interactions shared between the files was averaged for the 100 random data sets at each specified fraction of the total interactions sampled. The percentage to total was calculated from these averages and plotted as a collector's curve (Figure S5). Over 75% of the interactions present in each condition were detected after sampling just 10% of the total interactions. Therefore, we concluded that we have sampled a significant proportion of the interactions that were present in the *E. coli* cells at the time of cross-linking.

Quantification of the effects of bar-coding, sequencing and biological replicates on network patterns

The interactions within the biological repeats were highly correlated (figure S6). To further investigate the level of agreement between these samples, and the effects of sequencing on this agreement, we used Cohen's Kappa is a statistic that summarizes agreement in categorical variables. Binning the interaction counts into categories allows us to reflect the greater importance of concordance among interaction pairs with high counts. Cohen's Kappa ranges between 0 and 1 with 1 being perfect agreement; however, it is a conservative measure that controls for the level of chance matching expected. (See, e.g., (3)). Thus, the expected level of chance matching, controlled by the marginal probability of landing in each category, influences Kappa; we will study a case where these marginal probabilities are identical across the different situations of interest, so the Kappa measures can be directly compared.

To study the level of agreement between different sequencing results from different lanes, barcodes, and biological replicates, we have considered all interactions observed for a particular condition. The data have been modified to reflect how often each interaction occurs in each scenario, i.e., many counts of "0" have been added to show when an interaction is possible under a condition, but was not observed for a particular lane/barcode/replicate combination. For each combination of factors, we have then labelled each interaction as occurring in the bottom 95%, top 5% but not top 1%, top 1% but not top 0.5%, and top 0.5%. The top 5% typically starts at a count of around 5. The choice of these quantiles was based on the fact that lower counts have a large number of interactions with that count, thus the nominal proportion associated with the quantile can be misleading; e.g. if the median is 2, there may actually be 70% of the interactions with count ≤2. For the values chosen, the nominal proportions correspond well to the actual proportions, which are the marginal probabilities referred to above, enabling proper comparison of Kappa values. Kappa is defined for agreement between two variables, so all pairs of factor combinations are considered.

Barcoding, sequencing lane and biological replicates did not strongly affect the correlation between samples (Table S10). The differences introduced by different lanes and barcodes are indistinguishable from different barcodes alone; however, as expected, different biological replicates have lower agreement than those that differ only in barcode/lane (Table S10). The technical replicates (L1a, L1b, L1c and S1a, S1b, S1c) were highly correlated and were combined into L1 and S1 respectively. Following this pooling, Pearson's correlation analysis demonstrated that the biological replicates of the *E. coli* interaction networks were highly correlated at the HhaI restriction fragment level (exponential phase *E. coli* cells, r=0.879; and SHX treated cells r=0.935; Table S10). Thus the biological replicates (L1, L2 and S1, S2) were combined into exponential and SHX samples for the remainder of the analysis, respectively.

Filtering based on ligation controls

We wanted to know which of our individual interactions were above experimental noise. During the preparation of the GCC samples, random ligation events can occur during: 1) the GCC ligation step, and 2) the sequencing preparation step (i.e., the addition of linkers during sequencing library preparation). In an attempt to control for this, external controls were added during the GCC library

preparation to obtain estimates of the rates of inter-molecular ligation events. The three pUC19-gDNA ligation events with the highest interaction frequencies in both exponential and SHX treated samples were further assessed. The sequences of the *E. coli* fragments found to interact with pUC19 were extracted and fragmented using a 13 bp sliding window, shifted 1 bp at a time. These fragments were subsequently aligned to the pUC19 fragments they interacted with. If the pUC19 fragments and *E. coli* sequences aligned multiple times then the high level of interactions was attributed to a miss-alignment and not to a random ligation event. There was one *E. coli*-pUC19 interaction (frequency of 4) in the SHX treated sample that did not occur due to miss-alignment. Therefore, the cut-off for filtering out random ligations was set to 4 interactions; only fragment pairs that occurred 5 or more times were considered for analysis.

Analysis of loop size and interacting fragment distributions

Loop size calculations were performed on GCC interaction networks that had repetitive sequences and adjacent interactions removed. The size of the loop between two interacting DNA fragments was determined by taking the absolute value after subtracting the end position of the first interacting partner (having the smaller end coordinate of the two fragments) from the start position of the second interacting partner (having the larger start coordinate of the two fragments). Because the bacterial genomes used in this study are circular, if the loop size was greater than half the size of the genome than the loop size was subtracted from the total genome size to give the actual loop size. Size specified bins were used to count the number of loops that were of a particular size and the data was plotted as the number of loops versus the bin size. To determine the loop size as a function of the distance from the origin of replication, the distance of each interacting partner from the origin was calculated and the loop size was associated with the calculated distance. The loop sizes were binned and corrected for the number of loops per bin. The difference in the average loop size per bin between exponential and SHX samples was plotted.

Interaction frequency versus distance from the origin of replication

All GCC interaction network data or only long distance interaction network data (>800 bp) was used to calculate the interaction frequency as a function of the distance from the origin of replication. The distance from the origin of replication was calculated for each interacting partner and then the total interaction frequency for each interacting fragment was assigned to this particular distance from the origin. The data was binned (10,000 bp) and plotted as total interaction frequency per bin, corrected for the number of fragments per bin, versus the distance from the origin of replication.

Transcription microarray

E. coli strain CC72 (4) was grown in LB (Gibco, lot # 817849) at 37°C until the Optical Density (OD_{600}) reached 0.2. The SHX sample was treated at OD_{600} 0.2 with SHX (500µg/ml, 30min) prior to RNA isolation. RNA was isolated using the hot phenol procedure. Briefly, 800µl of cells were mixed with 700µl of 65°C phenol (pH 5.0) and 100µl of 16 x lysis buffer (320mM Na Acetate, 8% SDS, 16mM EDTA) and incubated (65°C, 5min). The RNA was extracted twice with phenol:$CHCL_3$:IAA (25:24:1, pH 8.0), precipitated with isopropanol and suspended in DEPC water. To remove the DNA, 40µg of RNA was treated with Turbo DNaseI (Ambion). The RNA was extracted twice with phenol:$CHCL_3$:IAA (25:24:1, pH 8.0), precipitated with isopropanol and suspended in DEPC treated water (Invitrogen). The cDNA library was constructed using the SuperScript Double-Stranded cDNA Synthesis Kit (Invitrogen) according to manufacturer's instructions and the cDNA was sent to Roche-Nimblegen for microarray hybridization. Each experiment (exponential or SHX) is a pool of three biological replicates. A total of two technical replicates were performed per condition (exponential and SHX).

The Expression values were generated using quantile normalization (5-7). The average expression level of each gene from the two biological replicates for exponential and SHX samples were calculated. To determine which genes were significantly up and down regulated in SHX treated compared to exponential samples the log2 of the SHX/exponential ratio was calculated. Genes were considered significantly up regulated if the log2 ratio was greater than +1.5 and the log2 of the raw expression level in SHX was greater than 9. In contrast genes were considered to be significantly down regulated if the log2 ratio was smaller than -1.5 and the log2 of the raw expression level in exponential phase was greater than 9 (Table S4).

Gene ontology (GO) term association analysis on the significantly up and down regulated genes

To determine whether the significantly up and down regulated genes were enriched for particular GO terms an R package called GOstats was used (8). The 'gene universe' contained all annotate genes from the *E. coli* gene products list (http://regulondb.ccg.unam.mx/data/GeneProductSet.txt) and was compared to the significantly up or down regulated gene sets (Table S4). A standard hypergeometric test was used with a p-value cut-off of <0.01 (Table S5).

Correlating transcription level with interaction frequency

The raw expression level of all genes as well as just genes with a high expression level (log2 of the raw expression level >9) was correlated with the total interaction frequency with these genes. Using the genomic coordinates of the genes obtained from a gene products list (http://regulondb.ccg.unam.mx/data/GeneProductSet.txt), the interaction frequency with each of these regions was determined by summing up the total interaction frequency within each region. Where the region overlapped with a restriction fragment the interaction frequency was proportionally assigned to the region depending on the percentage of overlap. The interaction frequency of each gene was then plotted against the raw expression level for each gene.

Transcription regulons

We wanted to determine whether the distribution of significantly up and down regulated genes was non-random across the genome relative to the origin of replication. The distance of each gene from the origin was calculated. The fold change in gene expression was binned (50,000 bp bins) according to the distance of the gene from the origin. The gene expression values were shuffled randomly 1,000 times. After each round of shuffling, a distance from the origin was assigned to each value and the values were binned as above. The expression level in each bin for the real data and the randomly generated data was corrected for the number of genes per bin. The average fold change in gene expression for the real data and randomly generated data was then plotted against the distance from the origin of replication. Additionally, an auto correlation analysis was performed in R using the non-binned expression data to determine whether the pattern of up and down regulated genes seen across the genome is random or not.

GC content and transcription level

The *E. coli* genome was fragmented using a 1,000 bp sliding window shifted 1 bp at a time and the percentage GC content of each fragment was calculated. The distance of each fragment from the origin of replication was calculated and the percentage GC content for each fragment was placed into the appropriate 50,000 bp bin. The average GC content per bin was calculated and plotted against the average fold change in gene expression per bin.

References

1. Rodley, C.D.M., Bertels, F., Jones, B. and O'Sullivan, J.M. (2009) Global identification of yeast chromosome interactions using Genome conformation capture. *Fungal Genetics and Biology*, **46**, 879-886.
2. Li, R., Li, Y., Kristiansen, K. and Wang, J. (2008) SOAP: short oligonucleotide alignment program. . *Bioinformatics Applications Note*, **24**, 713-714.
3. Landis, J.R. and Koch, G.G. (1977) The measurement of observer agreement for categorical data. *Biometrics*, **33**, 159-174.
4. Cagliero, C. and Jin, D.J. (2013) Dissociation and re-association of RNA polymerase with DNA during osmotic stress response in Escherichia coli. *Nucleic Acids Res*, **41**, 315-326.
5. Bolstad, B.M., Irizarry, R.A., Astrand, M. and Speed, T.P. (2003) A comparison of normalization methods for high density oligonucleotide array data based on variance and bias. *Bioinformatics*, **19**, 185-193.
6. Irizarry, R.A., Bolstad, B.M., Collin, F., Cope, L.M., Hobbs, B. and Speed, T.P. (2003) Summaries of Affymetrix GeneChip probe level data. *Nucleic Acids Research*, **31**, e15.

7. Irizarry, R.A., Hobbs, B., Collin, F., Beazer-Barclay, Y.D., Antonellis, K.J., Scherf, U. and Speed, T.P. (2003) Exploration, normalization, and summaries of high density oligonucleotide array probe level data. *Biostatistics*, **4**, 249-264.
8. Falcon, S. and Gentleman, R. (2007) Using GOstats to test gene lists for GO term association. *Bioinformatics*, **23**, 257-258.

Figures S1-S6

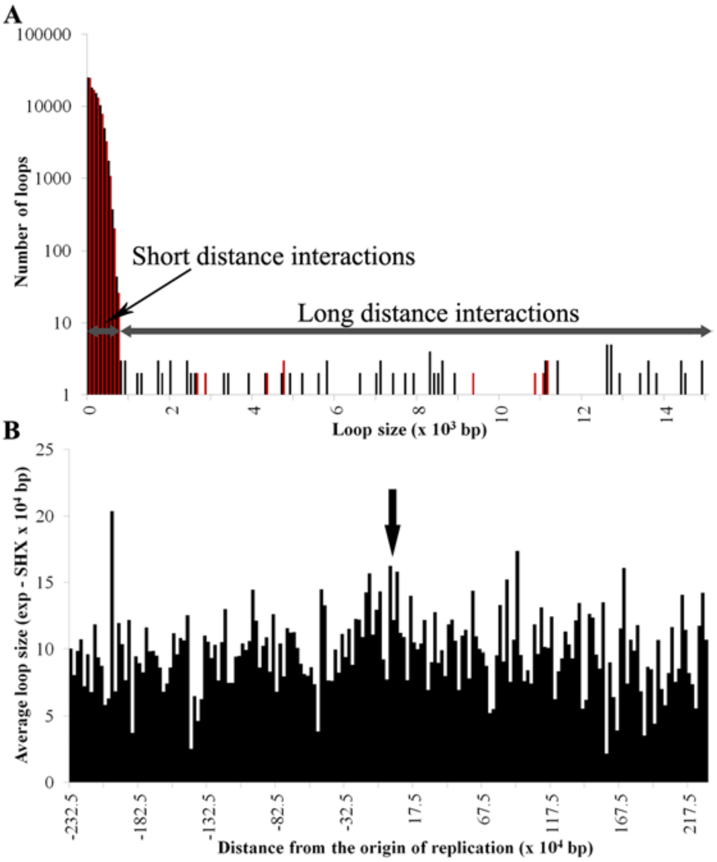

Figure S1. A) Interaction loop lengths within the *E. coli* genome can be divided into two distinct populations based on frequency: 1) Short (<800 bp) and 2) long distance (≥800 bp) interactions. The histogram of loop lengths (black, exponential phase; red, SHX treated) was calculated using 100 bp bins. For visualization, only loops less than 15 kbp in length have been shown. B) Loop lengths are longer in exponential phase cells than following SHX treatment (~98 kbp ± SE 2064). The difference between the average loop length for each pair of interacting fragments in exponential phase compared to the SHX treated cells was calculated and a histogram plotted using 25,000 bp bins centred at the origin of replication (3,923,883 bp). Since the *E. coli* chromosome is circular the loop size will always be less than half the length of the genome, therefore whichever of the calculated length (L_c) or L_r=4,639,675-L_c was shorter than 4,639,675/2 was counted for the frequency distribution. exp, exponential; SHX, serine hydroxamate. Origin position is indicated by the arrow.

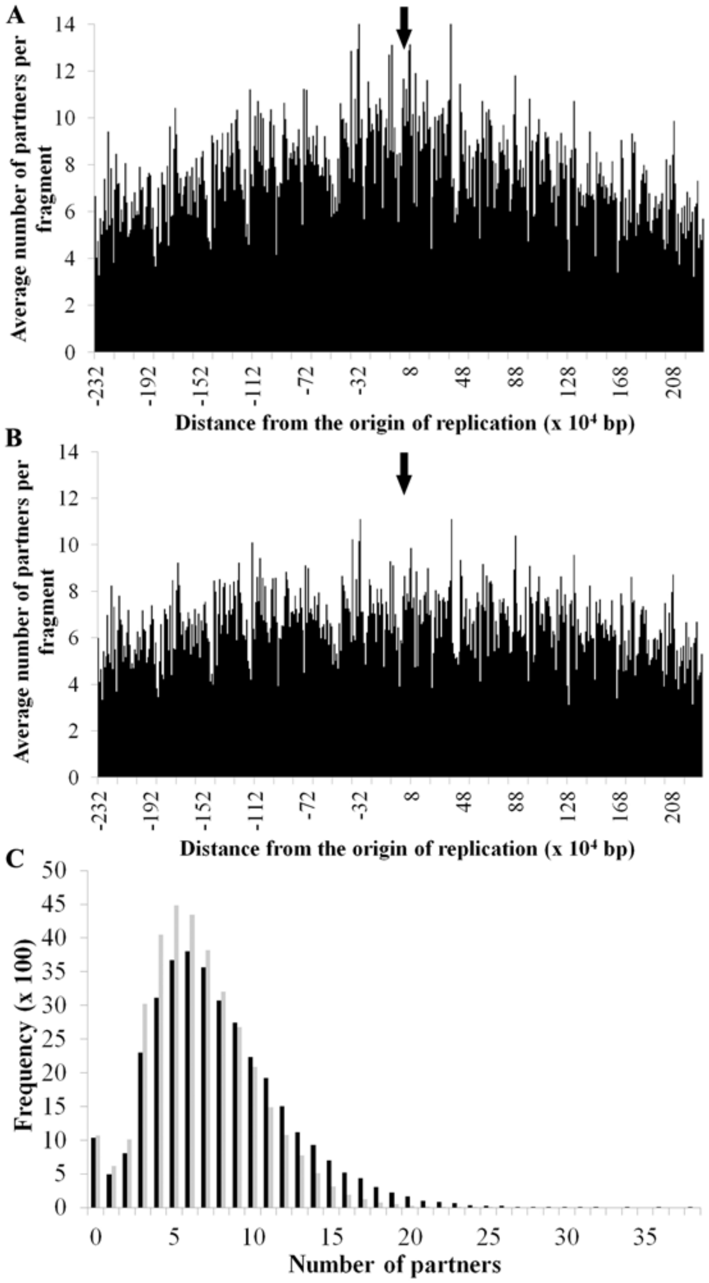

Figure S2. The distribution of the number of partners per restriction fragment across the *E. coli* genome averaged for 10,000 bp bins for exponentially grown A) and SHX treated B) cells. There is a visible increase in partner number towards the origin of replication (black arrow). C) There is no inter-condition difference in the mean number of unique partners per restriction fragment (exponential, black bars; SHX, grey bars). Adjacent and self-interactions were included in these calculations.

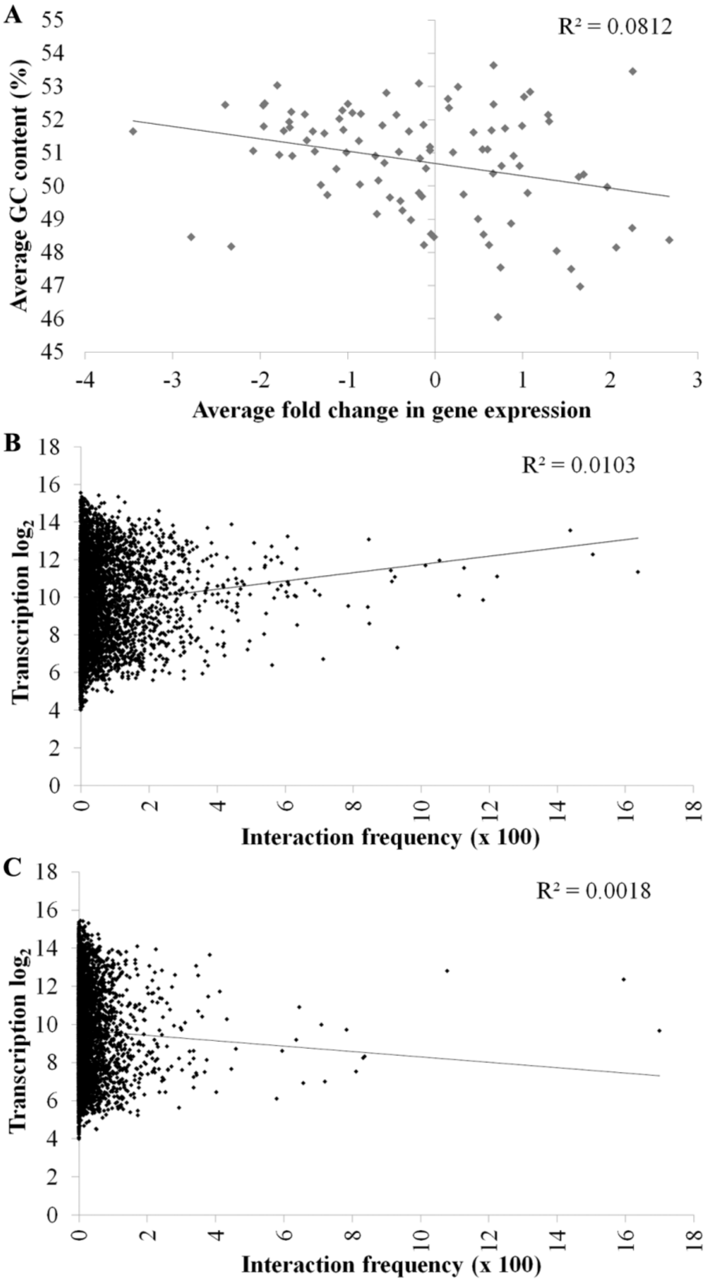

Figure S3. Genes whose transcript level changes upon SHX treatment did not correlate with genomic GC content and the raw transcript levels did not correlate with interaction frequency. A) Change in gene expression does not correlate with GC content. The genome was divided into 50 Kbp bins and the average change in gene expression and GC content calculated for each bin. The transcript levels (log2) of all annotated genes did not show any clear linear correlation with interaction frequency in both B) exponential phase and C) SHX interaction datasets (plots were generated from long distance interaction data [>800bp]).

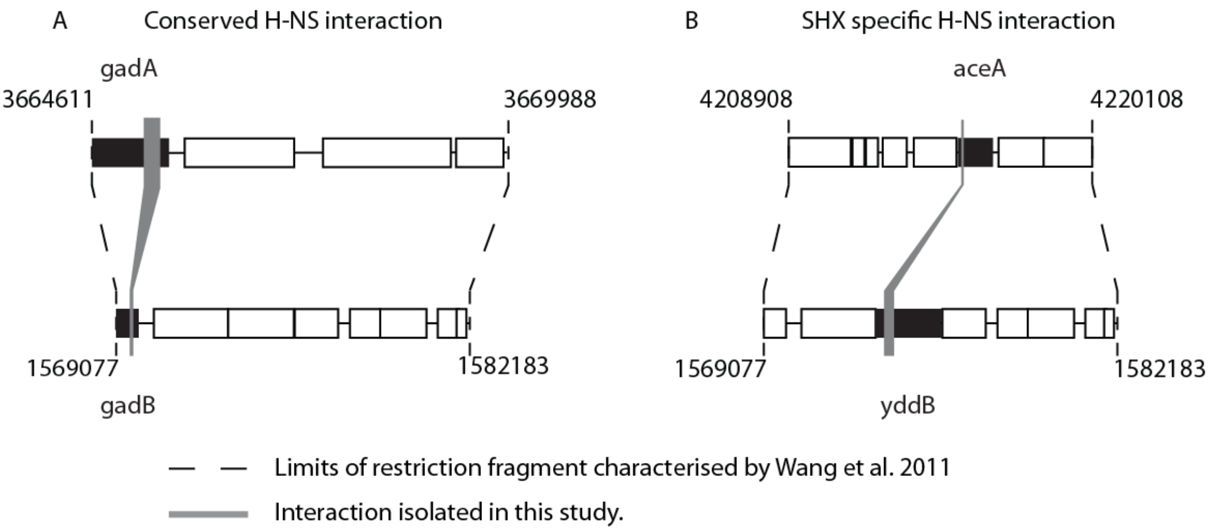

Figure S4. Interactions attributed to H-NS clustering identified by Wang et al. 2011 were confirmed as occurring between A) gadA:gadB and B) aceA:yddB (indicated by the grey bars linking the ORFs (black boxes)). The previously documented connections between the H-NS binding site containing gadA:ydeO and ydeO:arpA gene pairs occurred on large restriction fragments (illustrated as all of the DNA containing multiple ORFs occurring between the broken lines) that contained some H-NS binding sites and a mixture of H-NS regulated and non-regulated genes. Our interactions were fully contained within these large restriction fragments. The widths of the grey bars show the length of the restriction fragments that were characterized as interacting in this study.

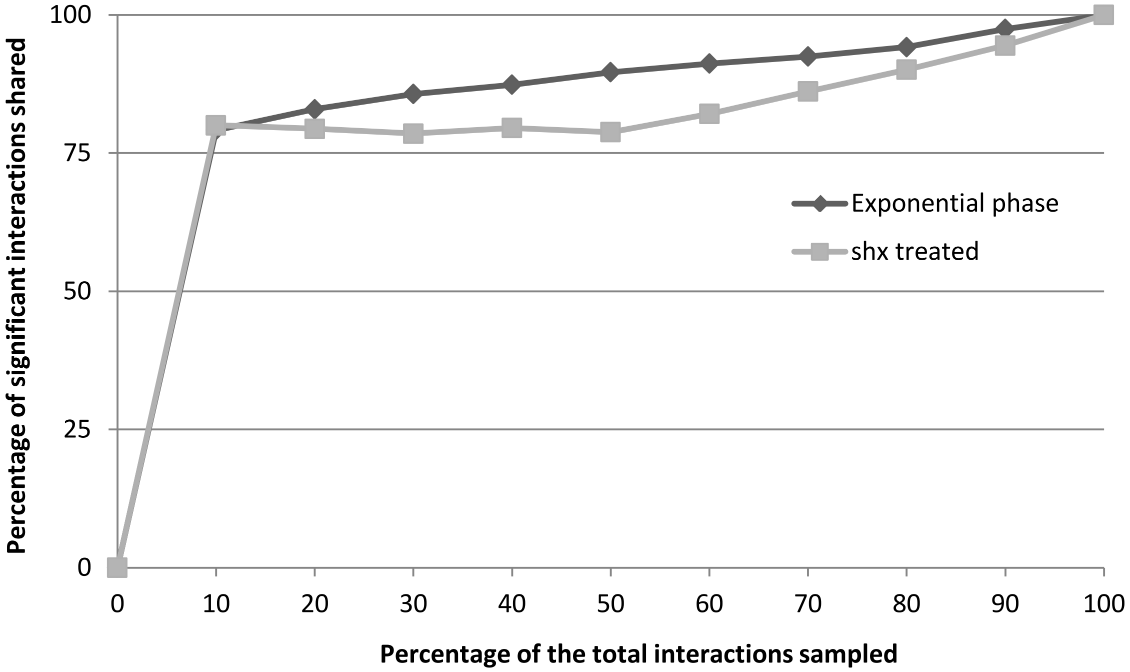

Figure S5. A collectors curve to determine the level of saturation of interaction detection. The average percentage of significant interactions in the random data sets that were also detected in the original data file was graphed. Over 75% of the interactions in the original data file were detected after sampling just 10% of the total interactions. We conclude that we have sampled a significant proportion of the interactions that were present in the populations of *E. coli* cells at the time of cross-linking.

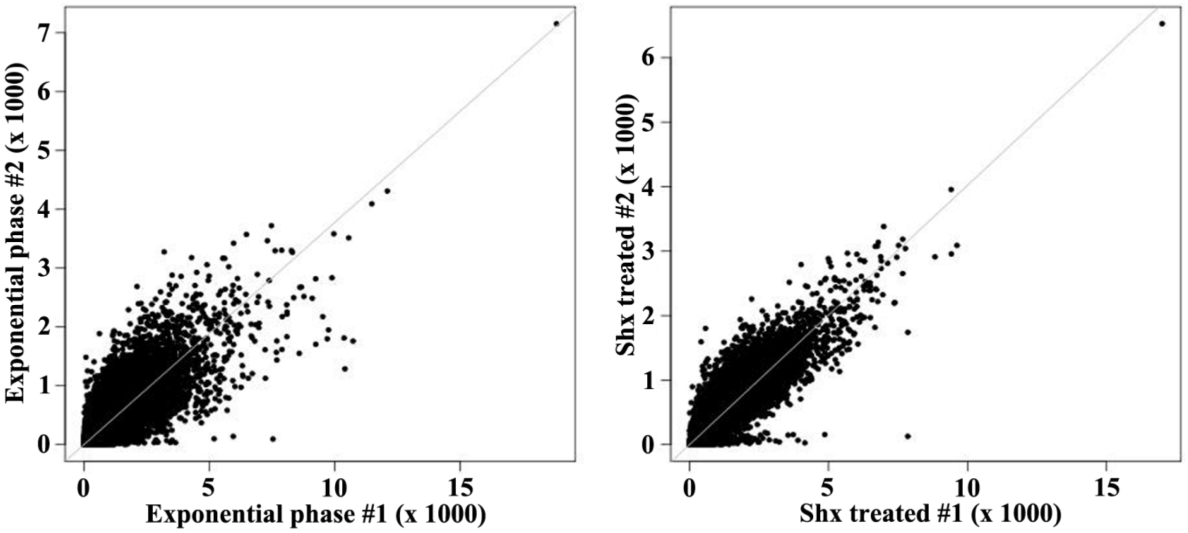

Figure S6. Biological replicates of the *E. coli* interaction networks were highly correlated at the HhaI restriction fragment level. Exponential phase *E. coli* cells (left, R^2=0.773) and SHX treated cells (right, R^2=0.8741). Scatter plots were constructed from all interactions in each dataset involving only HhaI fragments which could be uniquely positioned on the reference genome.

Tables S1-S10

Strain	Genotype	Reference
E. coli K12 CC72	F- lambda- *ilvG*- *rfb*-50 *rph*-1	(4)

Table S1. *Escherichia coli* strain used in this study.

Primer name	Sequence	Size
Lambda GCC F2	TGAAGAATGCCAGAGACTCC	
Lambda GCC R2	ACCCCGGTATCAGTTCATCC	
pRS426 GCC F2	AGTCACTGGCGCTTGGTCTGACAGTTACCAATGC	
pRS426 R GCC	GATAAATCTGGAGCCGGTGA	
Lambda control	TGAAGAATGCCAGAGACTCCGCTGAAGTGGTGGAAACCGCATTCTG TACTTTCGTGCTGTCGCGGATCGCAGGTGAAATTGCCAGTATTCTCG ACGGGCTCCCCCTGTCGGTGCAGCGGCGTTTTCCGGAACTGGAAAA CCGACATGTTGATTTCCTGAAACGGGATATCATCAAAGCCATGAACA AAGCAGCCGCGC	198bp
pRS426 control	GCGCTTGGTCTGACAGTTACCAATGCTTAATCAGTGAGGCACCTATC TCAGCGATCTGTCTATTTCGTTCATCCATAGTTGCCTGACTCCCCGTC GTGTAGATAACTACGATACGGGAGGGCTTACCATCTGGCCCCAGTG CTGCAATGATACCGCGAGACCCACGCTCACCGGCTCCAGATTTATC	187bp

Table S2. Ligation controls used in this study.

A

	Total number of unique interactions	Number of self-interactions	Number of adjacent interactions	Number of non-adjacent interactions
wt (LB)	148,036	21,369 (14.4%)	31,142 (21.1%)	95,525 (64.5%)
SHX treated	127,652	21,258 (16.6%)	30,972 (24.3%)	75,422 (59.1%)

B

	Break down of interactions shared by both condition				Break down of interactions specific to each condition			
	Total number of interactions	Number of self-interactions	Number of adjacent interactions	Number of non-adjacent interactions	Total number of interactions	Number of self-interactions	Number of adjacent interactions	Number of non-adjacent interactions
wt (LB)	117,174 (79.2%)	20,749 (14.0%)	30,710 (20.8%)	65,715 (44.4%)	30,862 (20.8%)	620 (2.0%)	432 (1.4%)	29,810 (96.6%)
SHX treated	117,174 (91.8%)	20,749 (16.2%)	30,710 (24.1%)	65,715 (51.5%)	10,478 (8.2%)	509 (4.9%)	262 (2.5%)	9,707 (92.6%)

C

	Exponential specific	Shared	SHX specific
Total (T)	30,862	117,174	10,478
Non-adjacent (NA)	29,810 (96.6% T)	65,715 (56% T)	9,707 (92.6% T)
>800 bp	17,512 (56.7% T) (58.7% NA)	1,838 (1.6% T) (2.8% NA)	6,275 (59.9% T) (64.6% NA)

Table S3. A summary of *E. coli* chromosomal interactions. A) Summary of interactions for *E. coli* cells growing in the exponential phase (wt (LB)) or following SHX treatment. B) Breakdown of the interactions in A) according to whether they were shared or unique for each condition. C) A further breakdown of the interactions specific to the exponential phase data set, shared by the two conditions or specific to SHX treated data set. In the condition specific interactions a large proportion of the non-adjacent interactions were long distance (>800bp), in contrast only a very small fraction of the shared interactions were long distance.

Up regulated	644

Down regulated	687

Table S4. The number of annotated *E. coli* genes that were significantly up or down regulated in response to SHX treatment.

GOBPID	P-value	OddsRatio	Expected Count	Count	Size	Term
**Genes up regulated in response to SHX treatment**						
GO:0016052	1.17378997e-06	2.38727829	31.04730473	56	274	carbohydrate catabolic process
GO:0051716	0.00041861	2.65896806	9.85808581	21	87	cellular response to stimulus
GO:0006950	0.00193190	2.20306513	12.46424642	23	110	response to stress
GO:0007154	0.00257035	4.30168350	2.60616062	8	23	cell communication
GO:0009432	0.00257035	4.30168350	2.60616062	8	23	SOS response
GO:0071496	0.00257035	4.30168350	2.60616062	8	23	cellular response to external stimulus
GO:0006281	0.00312075	2.95970009	5.09900990	12	45	DNA repair
**Genes down regulated in response to SHX treatment**						
GO:0034641	2.69704968e-10	2.38812533	76.95652174	121	402	cellular nitrogen compound metabolic process
GO:0009987	2.07328988e-08	2.39042032	299.9339934	338	1420	cellular process
GO:0009451	5.83763135e-08	5.40870332	9.50495050	26	45	RNA modification
GO:0009228	1.70810308e-06	21.1152815	2.74587459	11	13	thiamine biosynthetic process
GO:0042723	1.70810308e-06	21.1152815	2.74587459	11	13	thiamine-containing compound metabolic process
GO:0006259	9.18231075e-06	2.66460843	20.49609375	39	99	DNA metabolic process
GO:0009058	1.19923624e-05	1.64737243	145.5313531	182	689	biosynthetic process
GO:0006766	4.02275011e-05	3.71299775	9.08250825	21	43	vitamin metabolic process
GO:0044260	8.67573767e-05	1.64095992	98.0507974	127	500	cellular macromolecule metabolic process
GO:0044238	9.59316294e-05	1.66129574	273.7425743	303	1296	primary metabolic process
GO:0042559	0.00012636	4.89484030	5.28052805	14	25	pteridine-containing compound biosynthetic process
GO:0044271	0.00013130	1.78971560	52.0022309	75	252	cellular nitrogen compound biosynthetic process
GO:0006261	0.00018961	3.05169692	10.56105611	22	50	DNA-dependent DNA replication
GO:0009108	0.00063256	2.62343164	12.03960396	23	57	coenzyme biosynthetic process
GO:0019438	0.00070699	3.49461475	6.60345789	15	32	aromatic compound biosynthetic process
GO:0000270	0.00079379	2.70743802	10.77227723	21	51	peptidoglycan metabolic process
GO:0006022	0.00079379	2.70743802	10.77227723	21	51	aminoglycan metabolic process
GO:0006308	0.00095322	3.83018868	5.49174918	13	26	DNA catabolic process
GO:0018130	0.00130646	1.92686611	24.96932515	39	121	heterocycle biosynthetic process
GO:0055086	0.00158813	1.85590380	27.669967	42	131	nucleobase-containing small molecule metabolic process
GO:0006807	0.00176545	2.34938910	11.91571554	22	78	nitrogen compound metabolic process
GO:0006596	0.00231163	4.23351159	4.01320132	10	19	polyamine biosynthetic process
GO:0006760	0.00266277	6.63793103	2.32343234	7	11	folic acid-containing compound metabolic process
GO:0009246	0.00266277	6.63793103	2.32343234	7	11	enterobacterial common antigen biosynthetic process
GO:0046656	0.00266277	6.63793103	2.32343234	7	11	folic acid biosynthetic process
GO:0042364	0.00271803	3.02540557	6.67625899	14	32	water-soluble vitamin

						biosynthetic process
GO:0009057	0.00324070	2.43616438	10.34983498	19	49	macromolecule catabolic process
GO:0006575	0.00366576	4.278	3.59075908	9	17	cellular modified amino acid metabolic process
GO:0090304	0.00459034	1.7622705	25.68930892	38	131	nucleic acid metabolic process
GO:0009165	0.00568340	2.11795580	13.09570957	22	62	nucleotide biosynthetic process

Table S5. An R package for the analysis of Gene Ontology (GO) term associations called GOStats was used to determine significantly enriched GO terms in the up and down regulated gene sets. The table shows the significantly enriched GO terms in the up and down regulated gene sets.

		Clustering		Interactions	
NAP	Interaction set	RS	CLS	RS	CLS
matP	exp	High**	High**	NC	NC
	SHX	nd	nd	NC	NC

Table S6. The total number of interactions and clustering for matS sites (+/- 50 bp) was determined in the exponential and SHX treated interaction data sets. Significant clustering of the matS sites was detected in the exponential data set but no clustering was detected in the SHX data set. The total number of interactions was Not Changed (NC) relative to the random data sets. nd, not detected, **p<0.001.

A

		Clustering		Interactions	
NAP	Interaction set	RS	CLS	RS	CLS
SeqA	exp	High*	High	High**	High**
	SHX	High	High	High*	High*

B

SeqA locus			Interacting SeqA partner		
Gene	Start position	End position	Gene	Start position	End position
selB	3756040	3757884	ulaG	4416584	4416584
uraA	2616893	2618182	scpA	3058872	3058872
frlB	3499290	3500312	slt	4628756	4628756
kefA	485760	489122	ispG	2638708	2638708
gltL	683753	684478	ebgA	3220655	3220655
betA	324801	326471	yjfC	4411051	4411051
prpE	351930	353816	recB	2950483	2950483
galE	790262	791278	recB	2950483	2950483

C

SeqA locus			Interacting SeqA partners: First partner			Second partner			Third partner		
Gene	Start position	End position	Gene	Start position	End position	Gene	Start position	End position	Gene	Start position	End position
yhhJ	3623702	3624826	phnD	4321359	4321359						
hyfR	2609922	2611934	frlB	3499290	3499290						
sucA	757929	760730	smtA	972760	972760						
dmsA	940182	942626	ptsA	4137743	4137743						
potI	896307	897152	ptsA	4137743	4137743						
yghJ	3112572	3117134	frlB	3499290	3499290						
scpA	3058872	3061016	uxaA	3239849	3239849						
aaeB	3384243	3386210	dppA	3704121	3704121						
ypdE	2502532	2503569	parE	3171526	3171526						
betI	327971	328558	ybiW	859397	859397						
yghO	3127065	3128237	katG	4131858	4131858						
glpA	2350669	2352297	hda	2616097	2616097						
ybfF	711261	712025	uxaA	3239849	3239849						
prpE	351930	353816	maeB	2574120	2574120	ddlA	399053	399053	mukF	973542	973542
yfiQ	2717975	2720635	yghJ	3112572	3112572						
cusA	597937	601080	cysI	2886409	2886409	slt	4628756	4628756			

D

SeqA locus			Interacting SeqA partners: First partner			Second partner			Third partner			Fourth partner		
Gene	Start position	End position	Gene	Start position	End position	Gene	Start position	End position	Gene	Start position	End position	Gene	Start position	End position
yhhJ	3623702	3624826	psiE	4238348	4238348	hemN	4050068	4050068						
selB	3756040	3757884	psiE	4238348	4238348									
malZ	421739	423556	cusA	597937	597937									
araA	66835	68337	yahF	336002	336002									
hyfR	2609922	2611934	fadH	3229687	3229687									
nfrA	587205	590177	yjfC	4411051	4411051	nanA	3370705	3370705						
ebgA	3220655	3223747	dppA	3704121	3704121	pyrI	4469009	4469009						
ushA	504138	505790	smtA	972760	972760	mukF	973542	973542						
yahF	336002	337549	recB	2950483	2950483									
yfaL	2338439	2342191	scpC	3062824	3062824									
afuC	276980	278026	maeB	2574120	2574120									
scpA	3058872	3061016	ebgA	3220655	3220655	yjbH	4235657	4235657						
fryA	2500012	2502507	degS	3380222	3380222	ygiQ	3156949	3156949						
agaR	3275878	3276687	aceB	4213501	4213501									
hepA	60358	63264	sgrR	75644	75644	aaeB	3384243	3384243	scpC	3062824	3062824	hda	2616097	2616097
kefA	485760	489122	polA	4044989	4044989									
sgrR	75644	77299	afuC	276980	276980	uraA	2616893	2616893	hda	2616097	2616097			
gltL	683753	684478	mngB	767201	767201	slt	4628756	4628756						
rnd	1884888	1886015	ptsA	4137743	4137743									
betI	327971	328558	dnaA	3880349	3880349									
uxaA	3239849	3241336	phnD	4321359	4321359	kbaY	3281165	3281165						
yghO	3127065	3128237	sthA	4157413	4157413	frlB	3499290	3499290						
ybbO	517564	518373	ytfR	4449081	4449081									
hda	2616097	2616798	yjiN	4563989	4563989									
nanA	3370705	3371598	frlB	3499290	3499290	ptsA	4137743	4137743						
aroG	784856	785908	aslA	3982375	3982375									
glpA	2350669	2352297	pbpC	2643035	2643035									
betA	324801	326471	ybbO	517564	517564									
dinG	832293	834443	yjfC	4411051	4411051									
nrdB	2345406	2346536	ptsA	4137743	4137743									
prpE	351930	353816	cysI	2886409	2886409	ptsA	4137743	4137743	dnaC	4598261	4598261			
yfiQ	2717975	2720635	nfi	4196813	4196813									
pbpC	2643035	2645347	phnD	4321359	4321359									
miaB	692754	694178	scpC	3062824	3062824									

Table S7. SeqA sites cluster within the *E. coli* nucleoid. A) The total number of interactions and clustering for the strongest 135 confirmed SeqA binding sites was determined in the exponential and SHX treated interaction data sets. Significant clustering of the SeqA sites was detected in the exponential data set for the RS random sampling but not for the CLS random sampling and not in the SHX treated data set. The total number of interactions was significantly higher in both the exponential and SHX treated data sets. High p<0.1, *p<0.05, **p<0.01. B) Identities of the SeqA sites involved in clustering in the shared interactions (plotted in Figure 3C). C) Identities of the SeqA sites involved in clustering in the SHX specific interactions (plotted in Figure 3D). D) Identities of the SeqA sites involved in clustering in the exp specific interactions (plotted in Figure 3B). Clustering levels for B-D)

were calculated using non-adjacent interactions that occurred at levels higher than the false detection rate. Several SeqA loci were identified as being involved in interactions with multiple other SeqA loci (i.e. First-Fourth partners). The GCC assay does not allow us to determine if interactions with multiple other SeqA loci occurred at the same time within a single complex. Interactions are only shown in one direction (i.e. SeqA locus to partner 1 and not partner 1 to Seq A)

		Clustering		Interactions	
NAP	Interaction set	RS	CLS	RS	CLS
SlmA	exp	NC	NC	High*	High*
	SHX	NC	NC	High*	High

Table S8. The total number of interactions and clustering for the 24 defined SlmA binding sites was determined in the exponential and SHX treated interaction data sets. Clustering of the SlmA sites was Not Changed (NC) compared to random data sets in both the exponential and SHX treated data sets. The total number of interactions was significantly higher in both the exponential and SHX treated data sets apart from the CLS random sampling comparison for the SHX treated data set. High $p<0.06$, *$p<0.05$.

		Mean expression (per 20 kbp bin)		Expression variance (sum all bins)	
E. coli condition	Transcript N°. change on SHX shift	Test sample	Random sample (range)	Test sample	Random sample (range)
Exponential	Down	13,129 (High) $p = < 0.001$	6,910 – 10,337	225,684,597 $p = 0.113$	72,698,651 – 422,279,592
	Up	2,447 (Low) $p = < 0.001$	6,184 – 9,426	6,547,129 (Low) $p = < 0.001$	57,500,836 – 441,895,413
SHX	Down	2,271 (Low) $p = < 0.001$	6,464 – 9,992	9,293,946 (Low) $p = < 0.001$	58,259,459 – 372,181,089
	Up	12,733 (High) $p = < 0.001$	5,803 – 9,185	174,870,395 $p = 0.154$	41,320,084 – 388,277,906

Table S9. Genes that were highly expressed in exponential (exp) cells became weakly expressed in SHX treated cells and *vice versa*. The summed raw transcript level for the exp or SHX transcription microarray data for genes that were significantly up (SHX/exp log2 ratio >1.5) or down (SHX/exp log2 ratio <-1.5) regulated after SHX treatment was compared to that of 1,000 randomly generated data sets containing the same number and length (bp) elements as the test set in question. Annotated *E. coli* gene transcripts which showed log2 changes of >1.5 upon treatment with SHX were included in the analysis. SHX, serine hydroxamate.

	Condition	
Difference	exponential	SHX
Barcode only	0.73	0.70
Barcode and Lane	0.74, 0.74	0.69, 0.71
Barcode, Lane, Biological replicate	0.62, 0.62, 0.64	0.65, 0.68, 0.67

Table S10. Barcoding, sequencing lane and biological replicates did not affect the correlation between samples. We calculated Cohen's Kappa for agreement between each pair of datasets; the values are listed by the differing factors for that pair (i.e. differing sequencing lane only, differing barcode and lane, and differing biological replicate, barcode and lane; Supplementary methods).
